# The Subjective Experience of Dyspareunia in Women with Endometriosis: A Systematic Review with Narrative Synthesis of Qualitative Research

**DOI:** 10.3390/ijerph182212112

**Published:** 2021-11-18

**Authors:** Federica Facchin, Laura Buggio, Dhouha Dridi, Giussy Barbara, Paolo Vercellini

**Affiliations:** 1 Department of Psychology, Catholic University of the Sacred Heart, Largo Gemelli 1, 20123 Milan, Italy; 2 Gynaecology Unit, Fondazione IRCCS Ca’ Granda Ospedale Maggiore Policlinico, Via della Commenda 12, 20122 Milan, Italy; buggiolaura@gmail.com (L.B.); dh.dridi2@gmail.com (D.D.); giussy.barbara@gmail.com (G.B.); 3 SVSeD—Service for Sexual and Domestic Violence and Obstetrics and Gynaecology Emergency Department, Fondazione IRCCS Ca’ Granda Ospedale Maggiore Policlinico, Via della Commenda 12, 20122 Milan, Italy; 4 Department of Clinical Sciences and Community Health, University of Milan, Via Festa del Perdono 7, 20122 Milan, Italy; paolo.vercellini@unimi.it

**Keywords:** dyspareunia, endometriosis, qualitative research, subjective experience, systematic review

## Abstract

Although dyspareunia (pain during intercourse) is common in women with endometriosis, there is poor qualitative evidence describing women’s subjective experience of this symptom. This systematic review of qualitative research aimed to provide an in-depth exploration of women’s lived experience of dyspareunia (i.e., how they perceive and describe their pain, how they deal with it, how it affects their psychological health and intimate relationships). A total of 17 published articles were included. Our findings, derived from thematic analysis, highlighted that endometriosis-related dyspareunia manifests itself in multiple forms (deep, introital, and/or positional dyspareunia, at orgasm, during and/or after intercourse). Women use a variety of coping strategies to deal with sexual pain, such as interrupting or avoiding intercourse, enduring pain to seek pregnancy, and/or finding alternative ways to enjoy sexuality. Dyspareunia impairs women’s psychological health, especially in terms of poor self-esteem and sense of femininity and has negative consequences on intimate relationships. Unfortunately, both women and physicians are often reluctant to discuss sexual issues. Sexual health should be routinely assessed during counselling with endometriosis patients. Helping women find targeted strategies to enjoy sexuality despite endometriosis may significantly improve their psychological health and quality of life.

## 1. Introduction

Endometriosis is a chronic gynaecological disease characterized by the presence of ectopic implants of endometrial tissue (i.e., outside the uterus), with a consequent inflammatory condition [1,2]. Endometriosis affects approximately 8–10% of women of reproductive age (176 million women worldwide) and is associated with a variety of symptoms such as subfertility and different forms of pain (e.g., pain at menstruation (dysmenorrhea), chronic pelvic pain, pain at defecation (dyschezia), pain at urination (dysuria), and pain at intercourse (dyspareunia)) [3,4,5]. Besides dysmenorrhea, dyspareunia is one of the most common pain symptoms in women with endometriosis. In a study by Schneider et al. [1], 79% of adolescents and young adult women with the disease experienced dyspareunia, twice as often than adolescents and young adults without the condition (40%).

The huge negative impact of endometriosis on women’s quality of life (including work productivity) and psychological health is well known, especially due to the presence of severe pain symptoms and infertility [5,6,7,8]. Intimate relationships represent one of the most affected life domains [5], as recently underlined by Facchin et al. [9]. In this study, more than 60% of women with endometriosis who responded to an online survey reported that endometriosis had a negative impact on their past and current intimate relationships, which was significantly associated with dyspareunia (with r values ranging from 0.275 for past intimate relationships and 0.433 for the current relationship).

Quantitative research has provided evidence for the negative effects of endometriosis-related dyspareunia on women’s sexual quality of life, sexual functioning, and sexual and relational satisfaction, especially with regards to deep dyspareunia, which may also lead to reduced intercourse frequency and impaired psychological health, including self-esteem [2,10,11,12]. In a study by Agarwal et al. [5], a clinically significant reduction in the severity of dyspareunia among women with endometriosis was associated with health-related quality of life benefits, also in terms of improved sense of control and emotional wellbeing, self-image, social support, and sexual intercourse assessed using the Endometriosis Health Profile-30 questionnaire. In another study by Shum et al. [12], greater severity of deep dyspareunia was associated with impaired sexual quality of life, and this association remained significant after controlling for the effects of confounding factors such as introital dyspareunia, other types of pelvic pain symptoms, comorbidities (i.e., psychological and pain disorders), and demographic and behavioural factors.

## 2. Aims of this Review

Understanding how women with endometriosis subjectively experience and deal with dyspareunia is essential for healthcare providers, considering the remarkable impact of this type of pain on women’s lives and intimate relationships. Thus far, most research focused on quantitative aspects of sexuality in the context of endometriosis, whereas qualitative descriptions of women’s subjective experience of pain during sex were only included in a few studies [13]. It should also be considered that the most common self-report questionnaires used to assess sexual function in women with endometriosis (such as the Female Sexual Function Index and the Female Sexual Distress Scale) are not disease-specific, which further clarifies the importance of exploring how women experience dyspareunia focusing on their own words [13].

Qualitative systematic reviews may usefully contribute to the overall field of pain (including various forms of chronic pain, such as musculoskeletal pain) by clarifying the characteristics of people’s subjective experience: for instance, “what is it like to have chronic non-malignant pain”? [14], (p. 36). The importance of reviewing qualitative evidence in the specific context of endometriosis was underlined by Denny and Khan [15], who also provided suggestions for conducting systematic reviews of qualitative research using a rigorous methodology (i.e., clear research question, replicable systematic database search, explicit methods for quality appraisal and data synthesis).

The aim of this systematic review was to describe and understand women’s experience of endometriosis-related dyspareunia. For this reason, the focus of this study was on qualitative research, which is concerned with people’s subjective world [15]. Specifically, the research question was: what is it like to have dyspareunia for women with endometriosis (in terms of pain perceptions, coping strategies, impact on psychological health and quality of life, including intimate relationships)?

## 3. Materials and Methods

Because the PRISMA guidelines cannot be fully applied to systematic reviews of qualitative research, we referred to the ENTREQ (Enhancing transparency in reporting the synthesis of qualitative research) statement, which was developed to provide researchers and reviewers with a useful guidance to promote rigorous and comprehensive reporting of the synthesis of qualitative evidence [16]. A systematic literature search of two important health-related databases (Pubmed and PsycInfo) was performed in August 2021 (by F.F. and L.B.) using the following search terms, combined with Boolean operators: endometriosis AND (“qualitative research” OR “qualitative study” OR “grounded theory” OR “phenomenological study”). We used these broad search terms because we wanted to retrieve all the published qualitative endometriosis research, which is a small body of studies. The target population was represented by sexually active women with endometriosis, and our specific interest related to their subjective experience of dyspareunia as reported in studies using a qualitative design.

We included qualitative studies written in English and reporting evidence on dyspareunia in women with endometriosis. No time restrictions were applied, and we included all the relevant qualitative studies published until 30 August 2021 (date of last search). We excluded commentaries, literature reviews, doctoral theses, and qualitative studies reporting accounts exclusively from participants other than women with endometriosis (e.g., male partners or physicians). No attempt was made to retrieve unpublished material. The reference lists of the selected articles, as well as those of other literature reviews, were also examined to identify additional studies.

Duplicates were excluded from the analyses. Titles and abstracts were then screened, and the full text of the selected articles was examined. In case of articles reporting findings from the same sample, we chose the paper that provided the most relevant evidence to answer our research question. Full consensus was reached for all the studies included in this review.

### 3.1. Quality Assessment

The quality of the included studies was assessed independently by F.F. and D.D. using the Critical Appraisal Skills Programme (CASP) [17], as done in other reviews [18]. This appraisal tool is composed of 10 items aimed at evaluating the clarity and the relevance of the research question, the methodology used in the study, and the validity of the findings. Following the work of Gameiro et al. [18], the quality of each study was categorized as low, moderate, or high, when the study met ≤3, from 4 to 7, or ≥8 criteria, respectively. Disagreements between rates were solved with discussion until consensus was reached.

### 3.2. Data Extraction

Information regarding the authors, the period and the country of the study, aim, participants, data collection procedures, and data analysis was extracted from each article and organized in an Excel sheet by F.F. and D.D. In the same sheet we reported the prominent themes that appeared in each article with exemplificative quotations.

### 3.3. Data Synthesis

Considering the qualitative nature of the included studies and the specific focus of our review, the evidence provided by this body of literature was summarized using thematic analysis, following the guidelines provided by Dixon-Woods et al. [19]. This procedure, performed independently by F.F. and D.D., involved line-by-line reading and coding of the results section of each article to identify recurrent themes and group the evidence of the selected articles under thematic headings. Interview quotes derived from the included articles and related to each theme and subtheme were also reported, as done in other reviews of qualitative endometriosis research [20] and indicated in the ENTREQ statement [16].

## 4. Results

### 4.1. Description of the Included Studies

**Figure 1:**
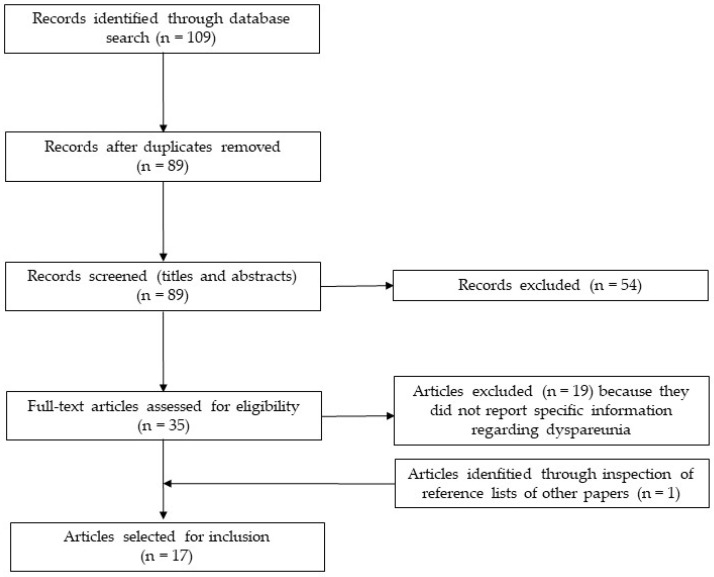
Selection process flowchart.

The selection process is represented in Figure 1. Of the 109 records initially identified through database search, 16 articles were included in this review. Fifty-four articles were excluded after screening titles and abstracts (reasons for exclusion were: not being a qualitative study [22 articles]; not being relevant [19 articles]; not including women with endometriosis, but other participants such as male partners or healthcare professionals [9 articles]; not being written in English [2 articles]; being a doctoral dissertation [2 articles]). The main reason for excluding full-text articles was the absence of information specifically related to dyspareunia. One additional article [21] was identified through the inspection of the reference list of other papers, therefore the final number of included qualitative studies was 17.

The characteristics of these 17 studies are summarized in Table 1. The number of women with endometriosis included in the studies ranged from 12 [22] to 74 [6], and the total number of participants was 456. Women’s age ranged from 17 [23] to 55 years [24]. Most of the participants had surgically diagnosed endometriosis. In the majority of the included studies, data were collected using semi-structured interviews, whereas focus-groups were conducted in only two studies [23,25]. Data analysis was performed using thematic analysis. A grounded theory approach was adopted in three studies [6,26,27], and phenomenology was used in other three studies [28,29,30]. Of the 17 included articles, only two [13,31] were specifically focused on endometriosis-related dyspareunia. The remaining 15 articles reported evidence derived from broader studies aimed at exploring the overall impact of endometriosis and its symptoms on women’s lives, or women’s vs. physicians’ perceptions of endometriosis (this specific research question was addressed by Fauconnier et al. [29] and by Riazi et al. [22]).

The results of quality assessment for the included studies are reported in Table 2. According to the quality ratings, there were no low-quality studies, six moderate- [21,22,24,25,26,28] and 11 high-quality studies [6,13,23,27,29,30,31,32,33,34]. Therefore, no studies were excluded from this review due to poor quality. The most important risks of bias were represented by an overall lack of consideration of the relationship between researchers and participants. For instance, none of the included studies systematically provided a reflection upon the researchers’ expertise and previous knowledge and understandings as a potential source of bias, which is important in qualitative research. In a few studies, especially those using a phenomenological approach, this issue was addressed in general, without providing a critical examination of the researchers’ specific role in the study [30].

### 4.2. Narrative Synthesis

In our review, data synthesis was performed using thematic analysis, which led to the extraction of five prominent themes entitled: (1) women’s perception of dyspareunia, (2) coping strategies, (3) psychological impact, (4) impact on intimate relationships, and (5) patient-provider communication. In this section, this body of evidence is summarized and presented using a narrative approach, with example quotations for each main theme derived from the published material examined. The themes identified for each of the included studies are reported in Table 1.

#### 4.2.1. Women’s Perceptions of Dyspareunia

This theme related to women’s subjective perceptions and descriptions of dyspareunia and was identified in 9 studies (53%) [13,21,22,23,26,27,29,31,32]. In a study by Fauconnier et al. [29], that included 41 patients with endometriosis, women talked about sex as an extremely painful experience, “*as if someone was sticking a needle*” in their pelvis, or as if their “*flesh was laid bare, a kind of electric feeling*” (p. 2689). Other words used by the patients to describe their pain were, for instance, “*sharp*”, “*burning*”, and “*deep*”. In a study by Wahl et al. [13], women’s qualitative descriptions of dyspareunia severity ranged from “*uncomfortable*” to “*the worst pain*” ever experienced, to the point that “*nothing could be done*” (p. 3). In a study by Denny [21], the pain experienced by one of the participants was so severe that she claimed: “*I am in agony*” (p. 645).

Pain can be experienced during and/or after intercourse (for hours or even for days) [27] and is mostly described as pain in the pelvis or pelvic organs (cramps, stabbing sensations, or severe convulsions), although women may have difficulties naming the specific site of their dyspareunia [13,26,29,31]. In the context of endometriosis, many women report pain at deep penetration, and/or painful penetration at the beginning of sexual intercourse (pulling, stinging, burning sensations), and/or pain at orgasm [13,32]. In some instances, pain is associated with specific sexual positions [13]. Pain following sex may be perceived as more tough to cope with than pain during intercourse [31]. In a study by Moradi et al. [23], one of the 35 women who participated in semi-structured focus-group discussions stated that after sex she would just “*lay down in a foetal position*” and cry for her pain (p. 4).

#### 4.2.2. Coping Strategies

The second theme was extracted from other 10 studies (59%) and described women’s strategies to deal with their pain [6,13,21,23,27,28,29,30,31,35]. Interrupting or completely avoiding sex due to pain, along with fear of painful intercourse, is common in women with endometriosis [6,21,23,29,35]. In a study by Wahl et al. [13], interruption of intercourse and avoidance of sex were reported by 88% and 59% of participants, respectively. The emotional burden of this situation was clearly described by Denny and Mann [31], who highlighted how tough it is for women who experience dyspareunia to engage in sexual intercourse with their loved one and then having to stop because of pain. Enduring pain during sexual intercourse is also common in women with endometriosis, especially among those who are seeking pregnancy [6,31]. A woman interviewed by Denny and Mann [31] stated that sometimes she just had “*to say no*”, and some other times she just had to “*suffer in silence*” (p. 191). Other strategies to deal with dyspareunia involve taking anti-inflammatories for the pain, using a hot water bottle, or having a shower [13]. Fortunately, some women can enjoy their sexuality despite endometriosis by finding an unpainful sexual position or identifying alternative ways to please themselves and their partner (i.e., other than vaginal penetration) [6,21,28,31].

#### 4.2.3. Psychological Impact

The negative impact of dyspareunia on multiple psychological dimensions (reflected by the third theme) has been highlighted in 11 studies (65%) [6,13,22,23,26,27,30,31,32,33,35]. In a grounded theory study by Facchin et al. [6], loss of interest in sex and complete avoidance of sexual intercourse was reported by women with clinically significant symptoms of anxiety and depression. As reported by Hållstam et al. [26], women may perceive their lives as ruined by endometriosis and its symptoms, including dyspareunia, which may lead to a subjective experience of existential grief due to physical limitations and missed opportunities in life, with increased dependence and feeling of being different. In this regard, dyspareunia can have a tremendous negative impact on women’s self-esteem and sense of femininity (especially in young women who ceased sexual activity), to the point that they may perceive themselves as “*half a woman*” [6,31]. Similarly, in the Moradi et al. study [23], women reported feelings of not being a woman due to impaired sexuality, with an overall negative body image due to weight gain and scars. “*I feel insignificant, you almost feel broken or something*”, claimed a woman interviewed in the study by Wahl et al. (13). Interestingly, Riazi et al. [22] highlighted the disruptive role of dyspareunia in their country (Iran) due to cultural pressures (i.e., being a wife and a mother to be a valuable woman). Comparisons with other women may also lead to feeling “*less womanly*” than women without endometriosis [35] (p. 909), along with feelings of inadequacy [27].

#### 4.2.4. Impact on Intimate Relationships

Evidence regarding the negative consequences of dyspareunia on intimate relationships was reported in 14 of the included articles (82%) [6,13,21,23,24,25,26,28,30,31,32,33,34,35]. Due to dyspareunia, sexual activity can be rare or even non-existent, leading to frustration and dissatisfaction in women, with negative consequences on their intimate relationships overall, also in terms of disruption of the couple’s plans for the future [33] and anxiety about initiating a new relationship [13,23,30,34]. Endometriosis-related sexual issues may also lead to tensions and conflicts within the couple, to the point of breaking up [25]. One of the main reasons for having sex despite dyspareunia is trying to get pregnant. In a study by Wahl et al. [13], one participant claimed: “*Lately, we just have sex because we want to conceive […] we don’t really have sex just because we want to*” (p. 4). Women may also feel obliged to have sex and thus endure pain to please their partner [24,28]. In addition, hormonal treatment leads to pain reduction on the one hand, but on the other hand it can cause loss of libido, which contributes to worsen the quality of couple intimacy [6,13]. Women can also experience feelings of shame and guilt towards their partner for avoiding sex and intimacy, along with regrets for missing out on sexuality [6,13,26,31].

In this complex scenario, partner support is essential. Women with good psychological health tend to perceive their partner as supportive and actively engaged in finding alternative solutions to enjoy sexuality as a couple, whereas those with worse psychological health often experience the sexual difficulties caused by endometriosis as their own problem (6). However, women often feel poorly supported and understood by their partner, who may minimize their pain, or even accuse them to be using pain to avoid sex, or to be having an affair [23,24,25,33,34]. In several studies, women also claimed that their partner felt rejected due to their avoidance of sex because of pain [28,31].

#### 4.2.5. Patient-Provider Communication

Surprisingly, information regarding communication between women and their doctors was provided in only two studies (12%) [21,28]. As reported by Denny [21], many women are reluctant to discuss issues related to their sexuality (including dyspareunia) with doctors, because this type of information is often considered too personal. This situation is further complicated by the fact that, in some instances, even doctors are reluctant and tend to minimize women’s pain by telling them that it is psychological: “*I was experiencing a lot of pain on penetration… I went to the doctors and they did an internal and said ‘Look, everything is perfectly normal’ and suggested that it might be a psychological problem, and I might just be anxious*” [21] (p. 645). In the study by Butt and Chesla [28], one woman (Emily) claimed that when she sought help from a provider she was simply recommended to use lubricants, which did not relieve her pain.

## 5. Discussion

The aim of this systematic review was to summarize the evidence provided by qualitative studies that reported data regarding women’s subjective experience of dyspareunia, a very common symptom of endometriosis experienced by more than half of women with the disease [36]. Consistently with the focus of our research question, the qualitative data analysed in the included studies were summarized using thematic analysis and presented with a narrative approach that also involved showing women’s own words (as reported by the authors of the included articles). At least to our knowledge, there are no other literature reviews specifically focused on women’s lived experience of endometriosis-related dyspareunia.

First, we clarified how women with endometriosis perceive and describe their dyspareunia (Theme 1), which is important to expand our knowledge (as researchers and clinicians) of their subjective experience. In this regard, Fauconnier et al. [29] compared patients’ descriptions of endometriosis-related symptoms with those provided by physicians and found that clinicians’ descriptions of dyspareunia were incomplete (i.e., did not capture all the themes described by the patients with regards to their own experience of pain). Our findings highlighted the variability in women’s subjective perceptions of dyspareunia and its multiple clinical manifestations, such as deep dyspareunia, introital/superficial dyspareunia, pain during and/or after intercourse, pain at orgasm, positional pain. Deep dyspareunia (i.e., pelvic pain experienced during deep vaginal penetration) is associated with infiltrating endometriotic lesions in the Pouch of Douglas, the uterosacral and cardinal ligaments, the posterior vaginal fornix and the anterior rectal wall [36,37]. Deep dyspareunia also depends on the presence of other comorbid conditions (either psychological or medical), such as depression, interstitial cystitis, painful bladder syndrome, myofascial pelvic pain syndrome, and central sensitization [12,36]. On the other hand, introital dyspareunia can be associated with provoked vestibulodynia or pelvic floor dysfunction [13].

Second, we described how women deal with endometriosis-related dyspareunia (Theme 2). In this regard, there is a variety of strategies: from dysfunctional strategies associated with poor psychological health (such as completely avoiding intercourse) and fear of painful sex, to functional strategies that involve for instance finding unpainful positions or alternative ways to enjoy sexuality (i.e., other than penetrative sex) [6,13]. Functional strategies to deal with dyspareunia are used by women with good mental health [6]. These data are useful to further understand the negative psychological impact of dyspareunia, which has also been highlighted in our review (Theme 3).

In this regard, we clarified that dyspareunia has a tremendous negative impact on women’s mood and especially self-esteem, along with feelings of guilt and shame towards the partner. Previous research demonstrated that sexual dysfunction and sexual distress in women with endometriosis are related to feelings of being an insufficient partner due to pain, physical tension during intercourse, fear of pain and enduring pain to please the partner [6,28,31], although trying to conceive remains the main reason for having sex despite dyspareunia [21,31]. The qualitative evidence summarized in this review also showed that endometriosis negatively impact intimate relationships (Theme 4), to the point of leading to relationship breakups, which has also been demonstrated in previous research [38]. In addition, some women receive poor support from their partner, who may even accuse them to have an affair [25].

Evidence regarding how doctors and patients communicate about dyspareunia and sexual issues (Theme 5) was reported in only two studies [21,28]. The poor available data suggest that the quality of patient-provider communication on sexual issues is low: both women and doctors are reluctant to discuss about sexual problems, and women who disclose their sexual difficulties may experience pain minimization (“it’s psychological”) [21] and receive inadequate suggestions with minimal solutions from doctors. As previously underlined by Vercellini et al. [39], women’s and doctors’ reticence to discuss about sexuality has led to an overall neglect of such an important endometriosis symptom, either in research or clinical practice.

### Limitations and Suggestions for Future Research and Clinical Practice

The small number of studies considered in this review confirms that women’s subjective experience of endometriosis-related dyspareunia remains a neglected topic, and represents a limitation of this review. The qualitative designs used in the included studies did not allow for generalizations of their findings, also considering the homogeneity of the samples (i.e., mostly young adult premenopausal women in heterosexual relationships). In addition, none of the included studies focused on adolescents, which limits our understanding of how dyspareunia affects the life of women at the very beginning of their sexual activity. It should also be considered that not all the 17 studies examined in this review included participants with surgically diagnosed endometriosis [13,26] or reported explicit information regarding diagnosis [24,28,30]. Indeed, the absence of surgical diagnosis did not allow for examining the association between endometriosis lesions and women’s experience of dyspareunia [13]. However, Agarwal et al. [4] recently underlined that diagnosing endometriosis using nonsurgical methods may have advantages, especially in terms of reduced diagnostic delays. The shift towards clinical diagnosis [13] is related to shifting the focus towards the patients (rather than the lesions), also considering that women’s subjective experience of pain may not be directly associated with the lesions [4].

The quality of the included studies was moderate or high, and the evidence summarized in this review allowed to depict a broad picture of women’s subjective experience of dyspareunia. However, some important endometriosis-related issues that are associated with dyspareunia were not or only marginally addressed. For instance, although there is evidence that women whose main motivation for having sex is to conceive are more likely to endure the pain during intercourse [40], the included studies did not directly examine the association between dyspareunia and infertility (which would be important, considering that approximately 50% of women with fertility problems have endometriosis [2]. In addition, superficial (rather than deep) dyspareunia can be associated with infertility concerns as reported by women with endometriosis [41]. This important issue should be addressed in future studies.

Patient–physician communication regarding dyspareunia is also a neglected topic. As recently reported by Witzeman et al. [42], this type of communication is often inefficient for several reasons. For instance, healthcare practitioners may be reluctant to discuss women’s sexual problems for fear of embarrassing the patients or themselves, or for religious beliefs. In general, there is a tendency to not routinely address sexual health issues, and women frequently receive incomplete information regarding their dyspareunia and how to deal with it. The findings reported in this review highlighted that helping women find targeted strategy to manage dyspareunia and thus improve their sexual health in the context of multidisciplinary practice would be extremely important [5]. Asking women to describe their pain may help clinicians clarify its causes (which may also be related to concurrent conditions such as provoked vestibulodynia or pelvic floor myalgia) and identify a personalized treatment approach, focused on women’s preferences and needs.

The quality of the communication between women and partners should also be explored in depth, because the available research evidence clearly indicates that partner support is essential, especially considering that many women tend to perceive dyspareunia as their own problem (rather than a shared problem). Engaging partners in endometriosis treatment, including psychological and sexological counselling, could improve their understanding of women’s suffering and the quality of the support provided [9,38].

## 6. Conclusions

Dyspareunia is a common symptom of endometriosis, but women’s subjective experience of sexual pain remains overlooked, especially with regards to patient–doctor communication. The evidence summarized in this review suggests that this form of pain may have a pervasive negative impact on women’s lives and deserves clinical attention. Women with endometriosis have the right to enjoy their sexuality and taking care of their sexual health should be routinely included in multidisciplinary clinical practice with our patients.

## Figures and Tables

**Table 1:** Characteristics of the included studies.

Article and Country	Aims	Participants	Women with Dyspareunia	Women’s Age Range	Type of Diagnosis	Data Collection	Data Analysis	Themes
Butt and Chesla, 2017 [28] (USA)	To examine the experience of couples living with endometriosis-related chronic pain	13 heterosexual couples	Not reported	23–48 years	Self-reported	In-depth individual and conjoint interviews	Thematic analysis; identification of exemplar and paradigm cases (interpretative phenomenology)	-Impact on intimate relationships -Patient-provider communication -Coping strategies
Denny, 2004 [21] (UK)	To investigate women’s experiences of living with endometriosis	15 women	12 women	20–47 years	Surgical	Narratively oriented semi-structured interviews	Thematic analysis, content analysis	-Women’s perceptions of dyspareunia -Impact on intimate relationships -Patient-provider communication -Coping strategies
Denny and Mann, 2007 [31] (UK)	To explore the impact of dyspareunia on women’s lives	30 women	23 women (of 27 sexually active women)	19–44 years	Surgical	Narratively oriented semi-structured interviews	Thematic analysis	-Women’s perceptions of dyspareunia -Psychological impact -Impact on intimate relationships -Coping strategies
Drabble et al., 2020 [32] (UK)	To investigate women’s experience of pain	20 women	14 women	21–over 51 years	Surgical	Semi-structured interviews	Thematic analysis	-Women’s perceptions of dyspareunia -Impact on intimate relationships -Psychological impact
Facchin et al., 2018 [6] (Italy)	To explain how and why endometriosis affects women’s psychological health	74 women	Not reported	24–50 years	Surgical	Open interviews	Grounded theory	-Psychological impact -Impact on intimate relationships -Coping strategies
Fauconnier et al., 2013 [29] (France)	To examine the descriptions of endometriosis-related symptoms as provided by patients vs. physicians	41 women	Up to 19 women raised themes related to dyspareunia	21–45 years	Surgical and clinical	In-depth interviews	Thematic analysis (phenomenological approach)	-Women’s perceptions of dyspareunia -Coping strategies
Hållstam et al., 2018 [26] (Sweden)	To investigate women’s experience of painful endometriosis and its treatment	13 women	Not reported	24–48 years	ICD10 code of N 801, 803, 808, or 809	Semi-structured interviews	Grounded theory	-Women’s perceptions of dyspareunia -Psychological impact -Impact on intimate relationship
Hudson et al., 2016 [33] (UK)	To explore biographical disruption in couples living with endometriosis	22 heterosexual couples	19 women	25–50 years	Surgical	In-depth semi-structured interviews	Thematic analysis	-Psychological impact -Impact on intimate relationships
Jones et al., 2004 [27] (UK)	To investigate the impact of endometriosis on quality of life	24 women	18 women	21.5–44 years	Surgical	In-depth interviews	Grounded theory	-Women’s perceptions of dyspareunia -Psychological impact -Coping strategies
Matías-González et al., 2020 [25] (Puerto Rico)	To investigate stigma experience in Latina women with endometriosis	50 women	Not reported	>21 years	Surgical	Focus-group	Thematic analysis	-Impact on intimate relationships
Moradi et al., 2014 [23] (Australia)	To examine the impact of endometriosis on women’s lives, also comparing three age groups	35 women	25 women	17–53 years	Surgical	Focus-group	Thematic analysis	-Women’s perceptions of dyspareunia -Psychological impact -Impact on intimate relationships -Coping strategies
Namazi et al., 2020 [34] (Iran)	To explore the impact of endometriosis among Iranian women	20 women	Not reported	23–43 years	Surgical	Semi-structured interviews	Content analysis	-Impact on intimate relationships
Rea et al., 2020 [30] (Italy)	To explore women’s lived experience of endometriosis	25 women	Not reported	18–54 years	Not reported	Open interviews	Cohen’s phenomenology	-Impact on intimate relationships -Psychological impact -Coping strategies
Riazi et al., 2014 [22] (Iran)	To explore patients’ and physicians’ experiences of occurrence and diagnosis of endometriosis	12 women	Not reported	22–37 years	Surgical	Semi-structured interviews	Content and thematic analysis	-Psychological impact -Women’s perceptions of dyspareunia
Roomaney and Kagee, 2018 [32] (South Africa)	To investigate health-related quality of life among women with endometriosis in South Africa	25 women	Not reported	25–42 years	Surgical	Semi-structured interviews	Thematic analysis	-Impact on intimate relationships -Psychological impact -Coping strategies
Seear, 2009 [24] (Australia)	To investigate stigmatizations, concealment of menstrual problems and diagnostic delay in women with endometriosis	20 women	Not reported	24–55 years	Not reported	Semi-structured interviews	Thematic analysis	-Impact on intimate relationships
Wahl et al., 2020 [13] (Canada)	To provide a qualitative description of women’s experience of endometriosis-related dyspareunia	17 women	17 women	23–50 years	Clinically suspected or diagnosed endometriosis	Semi-structured interviews	Thematic analysis	-Women’s perceptions of dyspareunia -Psychological impact -Impact on intimate relationships -Coping strategies

**Table 2:** Quality assessment of the included qualitative studies using the CASP Qualitative Research Checklist (Critical Appraisal Skills Programme; CASP 2013).

Study	1-Was There a Clear Statement of the Aims of the Research?	2-Is a Qualitative Methodology Appropriate?	3-Was the Research Design Appropriate to Address the Aims of the Research?	4-Was the Recruitment Strategy Appropriate to the Aims of the Research?	5-Was the Data Collected in a Way That Addressed the Research Issue?	6-Has the Relationship between Researcher and Participants Been Adequately Considered?	7-Have Ethical Issues Been Taken into Consideration?	8-Was the Data Analysis Sufficiently Rigorous?	9-Is there a Clear Statement of the Findings?	10-How Valuable is the Research?	Total
Butt and Chesla, 2007	Yes	Yes	Yes	Yes	Yes	No	No	Yes	Yes	Average	7 (Moderate)
Denny, 2004	Yes	Yes	Yes	Yes	Yes	No	Yes	No	Yes	Average	7 (Moderate)
Denny and Mann, 2007	Yes	Yes	Yes	Yes	Yes	No	Yes	No	Yes	High	8 (High)
Drabble et al., 2020	Yes	Yes	Yes	Yes	Yes	No	Yes	Yes	Yes	Average	8 (High)
Facchin et al., 2018	Yes	Yes	Yes	Yes	Yes	No	Yes	Yes	Yes	Average	8 (High)
Fauconnier et al., 2013	Yes	Yes	Yes	Yes	Yes	No	Yes	No	Yes	High	8 (High)
Hållstam et al., 2018	Yes	Yes	Yes	Yes	Yes	No	Yes	No	Yes	Average	7 (Moderate)
Hudson et al., 2016	Yes	Yes	Yes	Yes	Yes	No	Yes	Yes	Yes	Average	8 (High)
Jones et al., 2004	Yes	Yes	Yes	Yes	Yes	No	Yes	Yes	Yes	Average	8 (High)
Matias-Gonzalez et al., 2020	Yes	Yes	Yes	Yes	Yes	No	Yes	No	Yes	Average	7 (Moderate)
Moradi et al., 2014	Yes	Yes	Yes	Yes	Yes	No	Yes	Yes	Yes	Average	8 (High)
Namazi et al., 2020	Yes	Yes	Yes	Yes	Yes	No	Yes	Yes	Yes	Average	8 (High)
Rea et al., 2020	Yes	Yes	Yes	Yes	Yes	No	Yes	Yes	Yes	Average	8 (High)
Riazi et al., 2014	Yes	Yes	Yes	Yes	Yes	No	Yes	No	Yes	Average	7 (Moderate)
Roomaney and Kagee, 2018	Yes	Yes	Yes	Yes	Yes	No	Yes	Yes	Yes	Average	8 (High)
Seear, 2009	No	Yes	Yes	Yes	Yes	No	Yes	Yes	Yes	Average	7 (Moderate)
Wahl et al., 2021	Yes	Yes	Yes	Yes	Yes	No	Yes	Yes	Yes	High	9 (High)

For criteria 1 to 9: No = 0/Yes = 1; for criterion 10: Low/average = 0; High = 1; Total score (sum): 0–3 = Low; 4–7 = Moderate; 8–10 = High.

## Data Availability

The data generated in this study are included in this published article.
